# Atrial and Ventricular Strain Imaging Using CMR in the Prediction of Ventricular Arrhythmia in Patients with Myocarditis

**DOI:** 10.3390/jcm13030662

**Published:** 2024-01-23

**Authors:** Riccardo Cau, Francesco Pisu, Jasjit S. Suri, Gianluca Pontone, Tommaso D’Angelo, Yunfei Zha, Rodrigo Salgado, Luca Saba

**Affiliations:** 1 Department of Radiology, Azienda Ospedaliero Universitaria (A.O.U.), di Cagliari—Polo di Monserrato s.s. 554 Monserrato, 09045 Cagliari, Italy; fra.pisu1@gmail.com; 2 Stroke Monitoring and Diagnostic Division, AtheroPoint™, Roseville, CA 95661, USA; jsuri@comcast.net; 3 Department of Perioperative Cardiology and Cardiovascular Imaging, Centro Cardiologico Monzino IRCCS, 20138 Milan, Italy; gianluca.pontone@cardiologicomonzino.it; 4 Department of Biomedical Sciences and Morphological and Functional Imaging, G. Martino University Hospital, University of Messina, 98124 Messina, Italy; tommasodang@gmail.com; 5 Department of Radiology and Nuclear Medicine, Erasmus MC, Doctor Molewaterplein 40, 3015 GD Rotterdam, The Netherlands; 6 Department of Radiology, Renmin Hospital of Wuhan University, Hubei General Hospital, Wuhan 430064, China; zhayunfei999@126.com; 7 Department of Radiology, Universitair Ziekenhuis Antwerpen, 2650 Edegem, Belgium; r.salgado@outlook.com

**Keywords:** myocarditis, cardiovascular magnetic resonance, outcomes, ventricular arrhythmia, myocardial strain

## Abstract

(1) **Objective**: Myocarditis can be associated with ventricular arrhythmia (VA), individual non-invasive risk stratification through cardiovascular magnetic resonance (CMR) is of great clinical significance. Our study aimed to explore whether left atrial (LA) and left ventricle (LV) myocardial strain serve as independent predictors of VA in patients with myocarditis. (2) **Methods:** This retrospective study evaluated CMR scans in 141 consecutive patients diagnosed with myocarditis based on the updated Lake Louise criteria (29 females, mean age 41 ± 20). The primary endpoint was VA; this encompassed ventricular fibrillation, sustained ventricular tachycardia, nonsustained ventricular tachycardia, and frequent premature ventricular complexes. LA and LV strain function were performed on conventional cine SSFP sequences. (3) **Results:** After a median follow-up time of 23 months (interquartile range (18–30)), 17 patients with acute myocarditis reached the primary endpoint. In the multivariable Cox regression analysis, LA reservoir (hazard ratio [HR] and 95% confidence interval [CI]: 0.93 [0.87–0.99], *p* = 0.02), LA booster (0.87 95% CI [0.76–0.99], *p* = 0.04), LV global longitudinal (1.26 95% CI [1.02–1.55], *p* = 0.03), circumferential (1.37 95% CI [1.08–1.73], *p* = 0.008), and radial strain (0.89 95% CI [0.80–0.98], *p* = 0.01) were all independent determinants of VA. Patients with LV global circumferential strain > −13.3% exhibited worse event-free survival compared to those with values ≤ −13.3% (*p* < 0.0001). (4) **Conclusions:** LA and LV strain mechanism on CMR are independently associated with VA events in patients with myocarditis, independent to LV ejection fraction, and late gadolinium enhancement location. Incorporating myocardial strain parameters into the management of myocarditis may improve risk stratification.

## 1. Introduction

Myocarditis is characterized by inflammation of the myocardium, and is recognized for its heterogenous clinical presentation and outcomes [1,2,3,4]. The natural history of patients with myocarditis varies, and ranges from complete recovery to a spectrum of adverse cardiac complications including dilated cardiomyopathy, heart failure, recurrent myocarditis, ventricular arrhythmia (VA), and sudden cardiac death [4,5]. Various VAs, such as ventricular fibrillation, sustained ventricular tachycardia, nonsustained ventricular tachycardia, and frequent premature ventricular complexes, have been observed in patients with myocarditis and are linked to increased cardiovascular mortality [6]. This underscores the crucial need for effective risk stratification tools to guide physicians. Cardiovascular magnetic resonance (CMR) is a well-established non-invasive method for the diagnostic evaluation of patients with myocarditis [7,8,9,10]. It also offers valuable prognostic information [11,12,13]. Septal late gadolinium enhancement (LGE) has been identified as a predictor of adverse cardiovascular events, including VAs [14,15]. The recently proposed CMR-feature tracking can provide a sensitive, quantitative evaluation of myocardial contractility [16,17,18]. It enables the easy calculation of atrial and ventricular strain, without requiring additional sequences and it has been shown to increase diagnostic [19,20,21] and prognostic value in patients with myocarditis [22,23,24]. Doerner et al. demonstrated that combining atrial and ventricular strain parameters with the Lake Louise criteria can enhance the diagnostic performance in patients with myocarditis [19]. Regarding CMR-feature tracking’s prognostic value, Fischer et al. reported that global longitudinal strain represents an incremental and independent prognostic marker over clinical features and other CMR parameters, including LGE (HR: 1.21; 95% CI 1.08–1.36; *p* = 0.001) [25]. However, our current understanding of the significance of atrial and ventricular strain parameters on the occurrence of VAs in patients with myocarditis remains limited. CMR-feature tracking offers advantages such as not requiring contrast media administration. This makes it a viable option for patients with concomitant renal disease, allergies to gadolinium, or limited tolerance due to cardiac symptoms such as orthopnea [26,27,28,29]. Promising diagnostic possibilities are emerging with abbreviated CMR protocols that omit the use of contrast media [30,31,32,33]. Identifying predictive CMR parameters derived from an abbreviated CMR protocol is expected to unquestionably yield significant advantages in real-life clinical practice.

Therefore, the current study aimed to explore the potential value of atrial and ventricular strain parameters as supportive contrast-free CMR markers in predicting VAs in patients with myocarditis.

## 2. Materials and Methods

### 2.1. Study Population

In this retrospective, observational, single-center study, all consecutive patients with myocarditis who underwent CMR and fulfilled the modified Lake Louise criteria [10] between 3 March 2017, and 7 September 2023 were considered. Myocarditis was diagnosed based on the current recommendations of the Position Statement of the European Society of Cardiology Heart Failure Association [7].

Exclusion criteria included subjects <18 years old, previous myocardial infarction, pre-existing cardiomyopathy, and suspected or known prior irreversible myocardial damage.

Cardiovascular risk factors were collected from medical records. Hypertension was defined as a systolic blood pressure of ≥140 mmHg or a diastolic blood pressure of ≥90 mmHg at rest on more than two occasions, or the use of antihypertensive drugs [34]. Smoking status was defined as current smokers or never smokers. Cholesterol laboratory analyses were conducted following the standard in-house protocol. Diabetes status was assessed using the World Health Organization criteria [35] or an established diagnosis of type 2 diabetes. Obesity was defined as a BMI > 30, as defined by the World Health Organization criteria [36].

**Figure 1:**
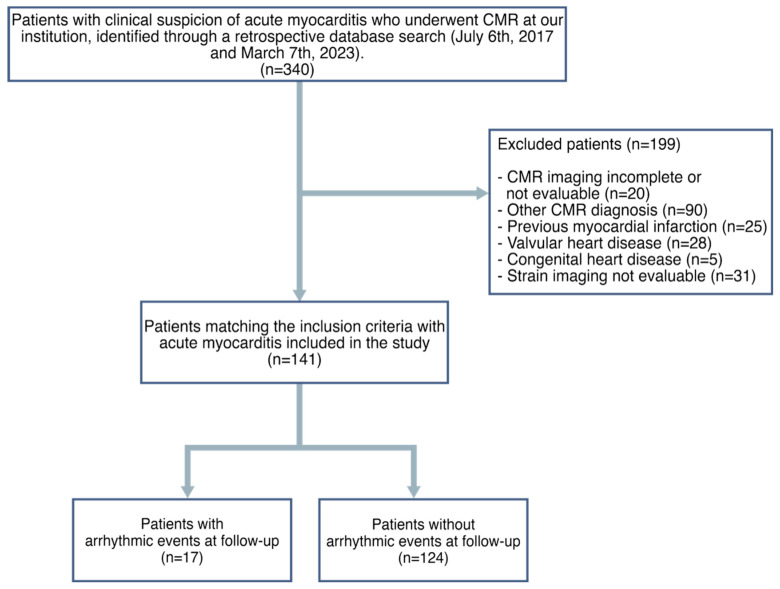
Flowchart of patients included in the study.

Institutional Review Board approval for this retrospective, cross-sectional study was obtained and the patient’s consent was waived given the retrospective nature of the study. A flowchart demonstrating the application of inclusion and exclusion criteria is provided in Figure 1.

### 2.2. CMR Acquisition

CMR examinations were conducted at 4.1 ± 2.6 days (median = 1 day, range = 1–10 days) post-hospital admission using a Philips Achieva dStream 1.5 T scanner system (Philips Healthcare, Best, The Netherlands) with anterior coil arrays. Cine-images were acquired using a balanced steady-state free precession and retrospective gating during expiratory breath-hold maneuvers (TE: 1.7 ms; TR: 3.4 ms/flip-angle: 45°, section thickness = 8 mm) in both long-axis (two-, three-, and four-chamber view) as well as in a short-axis plane, covering the entire ventricle from base to apex.

T2-STIR images were obtained using triple inversion recovery T2-weighted pulse sequence (TR = 2 RR, TE ≈ 70 ms; flip-angle: 45°, section thickness = 8 mm, FOV 300 × 300 mm^2^) in long-axis (two-, three-, and four-chamber view) and short-axis plane with whole ventricular coverage from base to apex.

T1 mapping was performed in the short-axis plane in three slices (at the base, mid-ventricular, and apex, respectively) using a single-breath-hold, ECG-triggered, MOLLI sequence before contrast media injection (TE 1.1 ms; TR 2.5 ms; flip angle 35°; FOV, 300 × 300 mm^2^).

T2 mapping was acquired before the administration of contrast-media on three representative short-axis slices (at the base, mid-ventricular, and apex, respectively) using a single-breath-hold, black-blood prepared ECG-triggered, spin-echo multiecho sequence.

LGE imaging was performed in both long- and short-axis slices 10–12 min after contrast media injection (Gadovist, Bayer Healthcare, Berlin, Germany) with a dose of 0.15 mL per kg body weight using phase-sensitive inversion recovery sequences (PSIR) (TE: 2.0 ms; TR: 3.4 ms; flip-angle: 20°, section thickness = 8 mm). The inversion time was determined using the Look-Locker technique.

### 2.3. CMR Image Post-Processing

We employed the commercially available software system Circle CVI42 (CVI42, Circle Cardiovascular Imaging Inc., Calgary, AB, Canada) for CMR-feature tracking analysis. Offline CMR-feature tracking analyses were performed to assess peak global longitudinal strain, global radial strain, and global circumferential strain in a 16-segment software-generated 2D model. For longitudinal strain, myocardial strain data were obtained from two-, three-, and four-chamber long-axis views. Concerning radial strain and circumferential strain, myocardial strain data were derived from apical, mid-ventricular, and basal short-axis views in all patients. On all images, the epi- and endocardial borders were delineated in end-diastole. Subsequently, an automated computation was initiated, whereby the applied software algorithm automatically traced the border throughout the entire cardiac cycle. Similarly, CMR-feature tracking analyses of atrial strain parameters were performed offline. On all the acquired images, LA endocardial borders were manually delineated in long view of the cine images when the atrium was at its minimum volume. The four-, three-, and two-chamber views were used to derive LA longitudinal strain. LA appendage and pulmonary veins were excluded from segmentation.

Consequently, the software algorithm effectively and accurately tracked the myocardium’s borders throughout the cardiac cycle using an automated computation process. Tracking and contouring quality were visually validated and manually corrected when necessary. Three peaks in the strain curve; namely, reservoir, conduit, and booster strain and their corresponding strain rate parameters were identified. The quality of the tracking and contouring of atrial and ventricular function was visually validated and manually corrected.

The extent and location of LGE was evaluated both qualitatively and quantitatively. Specifically, evaluation involved counting and determining the location of the affected myocardial segments. The extent of LGE were obtained by tracing the epicardial and endocardial contours in each short-axis image. A region of interest was manually positioned in normal myocardium, and LGE was defined as myocardium with mean signal intensity >5 standard deviations greater than the reference region of interest. All CMR parameters, including CMR-feature tracking, were analyzed by an operator blinded to patients’ baseline characteristics and outcomes.

### 2.4. Study End Points

Hospital medical records were meticulously reviewed to collect follow-up data. The primary outcome measured was the incidence of ventricular arrhythmia following CMR, including conditions such as ventricular fibrillation, sustained and nonsustained ventricular tachycardia, as well as frequent premature ventricular complexes.

### 2.5. Statistical Analysis

Continuous variables were presented as median (interquartile range [IQR]) or mean and standard deviation, while categorical variables were expressed as frequency (%). The normality of the distribution of the parameters was assessed using the Kolmogorov–Smirnov test. Comparisons of continuous variables were conducted through the independent samples *t*-test or Mann–Whitney U test, as appropriate. Categorical variables were analyzed using the chi-square test or Fisher’s exact test, as appropriate. Univariable analysis was performed using Cox proportional hazard regression to identify independent predictors of ventricular arrhythmia events. Atrial and ventricular strain predictors that demonstrated statistical significance (*p* < 0.05) during univariable analysis were subjected to further examination through multivariable Cox regression, adjusting for age, sex, common cardiovascular risk factors, left ventricular ejection fraction, and LGE septal location. To account for the influence of confounding factors, event-free survival from VA events at follow-up for continuous covariates that remained significant during multivariable analysis was calculated as a probability area using g-computation [37]. Changes in the hazard ratio across values of LV global circumferential strain—which demonstrated the highest hazard ratio following multivariable adjustment—were examined by fitting a spline curve. From this analysis, a cut-off of −13.3% was derived to stratify the population into low- and high-risk groups. This threshold corresponds to the point at which the hazard ratio reached or exceeded 1. All statistical tests were two-sided and a *p*-value < 0.05 was considered statistically significant. All statistical analyses were performed using R Statistical Software (v4.2.2; R Core Team 2022, Vienna, Austria).

## 3. Results

### 3.1. Patient Population

During the study period, a total of 141 patients (29 females, mean age 41 ± 20 standard deviation) with myocarditis were enrolled after the application of inclusion and exclusion criteria. (Figure 1). Baseline characteristics of patients are shown in Table 1. During a median follow-up of 23 months (IQR [18,19,20,21,22,23,24,25,26,27,28,29,30]), 17 patients (12%) had a VA event (14 males, mean age 59 ± 14), while 124 patients (88%) either completed the follow-up period event-free or were censored (age 39 ± 19).

### 3.2. Associations of Ventricular and Atrial Strain Measures with Ventricular Arrhythmia Risk

**Figure 2:**
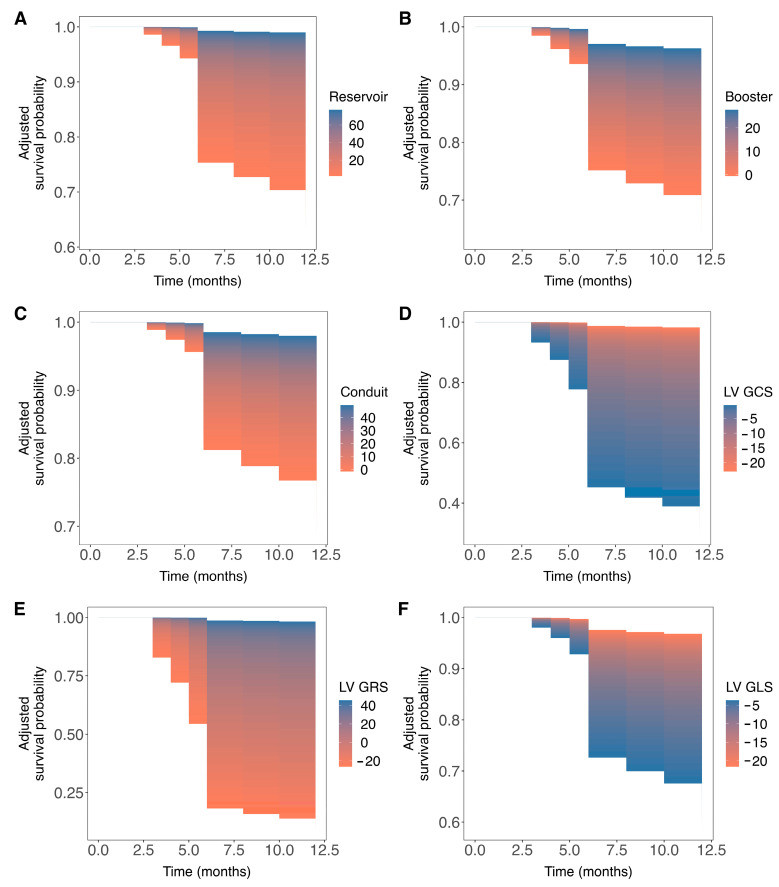
Event-free survival probability by prognosticators values during follow-up. Panels (**A**–**F**) display three-dimensional survival areas for left atrial (LA) reservoir, LA conduit, and LA booster strain measures and global measures of left ventricular (LV) circumferential, LV radial, and LV longitudinal strain illustrating the arrhythmia-free survival probability (y-axis) at various time points during follow-up (x-axis) up to 12 months across a range of prognosticators values (color-coded). For instance, panel (**A**) demonstrates a higher probability of event-free survival within 12 months for higher LA reservoir values, while panel (**D**) correlates higher LV global longitudinal strain with improved event-free survival outcomes.

Univariable Cox regression analysis revealed that increased weight, the presence of hypertension, dyslipidemia, diabetes mellitus, and diminished LA reservoir, LA conduit, LA booster, and LV global radial strain values were significantly associated with an increased incidence of VA events during follow-up. Additionally, increased LV end-diastolic and end-systolic volumes indexed by body surface area, impaired LV global longitudinal and circumferential strain values were also significantly associated with an increased risk of VA events (Table 2). Subsequent multivariable analysis identified LA reservoir (HR: 0.93 95% CI [0.87–0.99], *p* = 0.02) and booster (HR: 0.87 95% CI [0.76–0.99], *p* = 0.04) functions, as well as LV global longitudinal (HR: 1.26 95% CI [1.02–1.55], *p* = 0.03), circumferential (HR: 1.37 95% CI [1.08–1.73], *p* = 0.008) and radial (HR: 0.89 95% CI [0.80–0.98), *p* = 0.01) strain measures as statistically significant, independent predictors of VA events after adjustment (Table 3). Except for elevated LV global circumferential and longitudinal strain, decreased values in all other strain measures were associated with an increased risk of VA events at follow-up (Figure 2).

**Figure 3:**
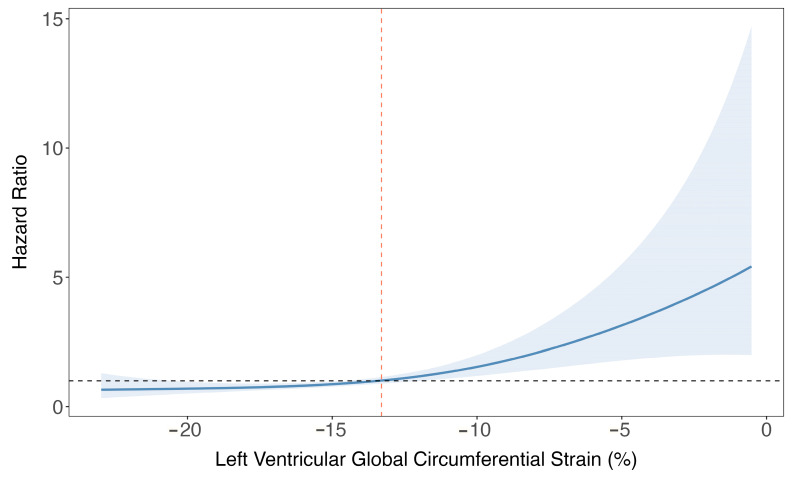
Spline curve showing the hazard ratio for the occurrence of ventricular arrhythmic (VA) events at follow-up according to left ventricular global circumferential strain (LV GCS). This curve (blue line) shows the association of hazard ratio for the incidence of VA events along with 95% confidence intervals (blue bands) across a range of values of LV GCS at the time of the cardiac MR. The value of LV GCS in which the predicted HR is ≥1 can be used as a cut-off to stratify the population in high- and low-risk (dashed orange line).

**Figure 4:**
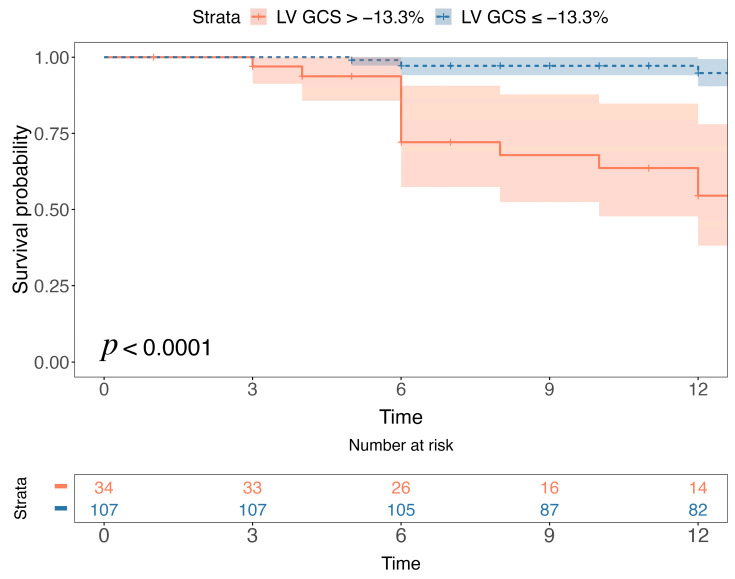
Arrhythmia-free survival analysis according to left ventricular global circumferential strain (LV GCS) > −13.3% vs. GCS ≤ −13.3%. Kaplan-Meier curves for the occurrence of ventricular arrhythmic (VA) events at follow-up show that patients with LV GCS > −13.3% are more likely to experience VA events.

Stratification of the population in low- and high-risk groups, based on a cut-off value of −13.3% for LV global circumferential strain (Figure 3), demonstrated that patients with an LV global circumferential strain higher than −13.3% experienced significantly worse event-free survival (*p* < 0.0001) (Figure 4).

## 4. Discussion

The current study evaluated the prognostic significance of atrial and ventricular myocardial strain in patients with myocarditis. Our findings reveal that both atrial and ventricular strain parameters are independently associated with VA events in myocarditis patients. Significantly, these myocardial strain indices maintain their predictive power independently of established CMR risk factors such as left ventricular ejection fraction (LVEF) and the location of LGE. Among these parameters, LV circumferential strain showed the most significant correlation with the incidence of VA events during the monitoring period.

Patients suffering from myocarditis can experience VAs with an annual rate of 10%, while the percentage of myocarditis-related sudden cardiac deaths attributed to myocarditis varies between 10% and 20% during autopsy [14]. Therefore, identifying patients at increased risk of VAs is crucial for accurately managing myocarditis cases. The location of myocardial replacement fibrosis detected through CMR-LGE has been established as an independent predictor of VA events in myocarditis patients [12,14,38,39]. In a study involving 144 patients with a history of myocarditis, Casella et al. reported that the location of LGE scar was an independent predictor of VA (HR 2.0; 95% CI 1.2–3.5; *p* = 0.02) regardless of treatment strategy [14]. Indeed, myocardial replacement fibrosis signifies both irreversible myocardial damage and an arrhythmic substrate [8]. In clinical practice, the adoption of faster and more cost-effective CMR protocols offers undeniable benefits. This approach enhances the accessibility of CMR examinations, making them available to a broader range of patients, including those who are unable to receive contrast agents and have a reduced tolerance for lengthy procedures. Consequently, there is a growing incentive to identify alternative markers that do not require contrast administration but can still effectively enhance the prediction of VA events.

Myocardial strain is intricately linked to the structural characteristics of the heart muscle fibers. It can quantitively detect early-stage impairments in both atrial and ventricular function [16,40,41] and it is related to cardiovascular complications [25,42,43,44]. In addition, strain parameters can be measured on standard cine sequences without the necessity for additional acquisitions or contrast media administration.

Atrial and ventricular strain parameters have proven to be robust markers of future arrhythmia events in various cardiovascular diseases [45,46,47,48]. Ersbøll et al. conducted a prospective study investigating the utility of global longitudinal strain in predicting VAs in the acute phase of myocardial infarction. The study revealed a significant reduction in longitudinal strain among patients who developed VAs in comparison to those who did not (9.9% vs. 13.9%, *p* < 0.001). Diminished longitudinal strain emerged as an independent predictor of VAs, even after accounting for various clinical, electrocardiographic, and echocardiographic factors [45]. Candan et al. assessed the effect of atrial strain in predicting sudden cardiac death or ventricular arrhythmias in patients with hypertrophic cardiomyopathy. The authors reported that atrial strain was an independent predictor of appropriate implantable cardioverter defibrillator therapy (odds ratio: 0.806, *p* = 0.008) [48]. To the best of our knowledge, this is the first work specifically focused on investigating the prognostic role of CMR-derived atrial and ventricular myocardial strain in patients with myocarditis.

Our data support the hypothesis that both atrial and ventricular strain are related to VA events. One of the key findings in the present study is that LV circumferential strain is independently associated with VA events in myocarditis and it can be clinically used to discriminate high-risk myocarditis patients. The significance of LV circumferential strain as a predictor of VA has been previously described in patients with hypertrophic cardiomyopathy [49] and ischemic cardiomyopathy [50]. The myocardium of the ventricular wall is organized into three layers of fibers: subendocardial fibers, subepicardial fibers, and transmural fibers [16]. LV circumferential strain, reflecting circumferential shortening, is predominantly influenced by changes in subepicardial fibers [16]. The heightened impact of subepicardial fibers as a predictor of VAs may be related to the typical myocardial fibrosis pattern observed in myocarditis [8], leading to electric remodeling [51]. Recent studies have shown that the subepicardial myocardium is of great significance in the occurrence of VAs [52]; this supports the concept that arrhythmic events in myocarditis may be related to re-entry circuits within the subepicardial layers [51]. Of interest, LA reservoir and booster parameters are independently associated with VAs at follow-up. This association may arise from the anatomical communication between cardiac chambers. The LA actively modulates left ventricular filling through its distinct phases [17,40,53]. Another hypothesis is that the direct involvement of the atria during myocarditis contributes to proarrhythmogenic remodeling of the atria [54]. Our results demonstrate that utilizing atrial and ventricular strain serves as helpful and supportive non-contrast CMR parameters for risk stratifying patients with myocarditis at risk of arrhythmic complications.

This study has certain limitations. Firstly, due to its retrospective design, some clinical and laboratory data were not available for analysis in every patient. Secondly, the relatively modest sample size, coupled with a limited number of events, may have increased overfitting risk in our multivariable analysis. Therefore, information on incremental prognostic value of the models is limited. Moreover, it is important to note that our investigation did not incorporate the use of continuous arrhythmia monitoring via implantable devices; this constrained our ability to capture real-time data on specific events. Additionally, endomyocardial biopsy or genetic testing to rule out alternative diagnoses was not conducted in all patients, despite this being common clinical practice in many centers currently [15,22]. Furthermore, the absence of a dedicated validation set also warrants caution in applying our findings to a broader population. Although our study yielded promising results, it is imperative to conduct additional prospective trials with a larger patient cohort to validate our findings.

## 5. Conclusions

LA and LV strain parameters are independently associated with ventricular arrhythmia, independently of cardiovascular risk factors, LV systolic function, and LGE location. Atrial and ventricular strain may be used as additional non-contrast CMR parameters to stratify risk in patients with myocarditis. The current findings may have a substantial influence on clinical decision-making and contribute to tailored care in this category of patients.

## Figures and Tables

**Table 1:** Baseline and CMR characteristic of patients with and without ventricular arrhythmia events. Values are *n* (%) or median (IQR); *p*-values in bold are significant. BSA indicates body surface area; LVEF and RVEF, left and right ventricular ejection fraction, respectively; EDV, end-diastolic volume; ESV, end-systolic volume; SV, stroke volume; GRS, GCS, and GLS, global radial circumferential and longitudinal strain, respectively; LGE, late gadolinium enhancement.

Variable	Overall, *n* = 141 ^1^	Event, *n* = 17 ^1^	No Event, *n* = 124 ^1^	*p*-Value ^2^
Gender (male)	112 (79%)	14 (82%)	98 (79%)	>0.99
Age, years	40 (22, 56)	56 (50, 70)	37 (21, 52)	**<0.001**
Height, cm	170 (170, 175)	170 (168, 175)	170 (170, 175)	0.89
Weight, kg	72 (63, 82)	80 (72, 90)	70 (60, 79)	**0.020**
BSA, m^2^	1.86 (1.72, 1.98)	1.88 (1.76, 2.05)	1.82 (1.69, 1.94)	0.25
Hypertension	26 (18%)	7 (41%)	19 (15%)	**0.018**
Dyslipidemia	16 (11%)	5 (29%)	11 (8.9%)	**0.027**
Obesity	16 (11%)	5 (29%)	11 (8.9%)	**0.027**
Current or previous smoking	20 (14%)	2 (12%)	18 (15%)	>0.99
Diabetes mellitus	6 (4.3%)	3 (18%)	3 (2.4%)	**0.023**
Family history of coronary disease	30 (21%)	4 (24%)	26 (21%)	0.76
Chest pain	125 (89%)	9 (53%)	116 (94%)	**<0.001**
Heart failure	11 (7.8%)	4 (24%)	7 (5.6%)	**0.029**
Arrhythmias	14 (9.9%)	10 (59%)	4 (3.2%)	**<0.001**
Reservoir, %	30 (24, 38)	20 (10, 29)	30 (25, 39)	**<0.001**
Conduit, %	18 (12, 23)	8 (5, 15)	18 (14, 23)	**<0.001**
Booster, %	12.2 (9.8, 16.0)	11.2 (7.2, 14.3)	12.8 (10.1, 16.0)	0.10
LVEF, %	56 (50, 61)	49 (41, 58)	57 (52, 61)	**0.022**
LV EDV/BSA, mL/m^2^	92 (80, 103)	96 (84, 129)	91 (80, 102)	0.054
LV ESV/BSA, mL/m^2^	40 (32, 48)	51 (41, 75)	39 (32, 45)	**0.023**
LV SV/BSA, mL/m^2^	51 (45, 57)	47 (42, 52)	52 (46, 57)	0.22
RVEF, %	55.7 (52.0, 58.7)	51.3 (49.2, 58.9)	55.9 (52.4, 58.6)	0.17
RV EDV/BSA, mL/m^2^	82 (71, 95)	81 (72, 88)	82 (71, 96)	0.68
RV ESV/BSA, mL/m^2^	35 (30, 43)	36 (29, 44)	35 (30, 43)	0.73
RV SV, mL/m^2^	46 (38, 53)	39 (37, 47)	46 (40, 53)	0.082
LV GRS, %	22 (18, 29)	16 (13, 20)	23 (19, 29)	**<0.001**
LV GCS, %	−14.4 (−17.0, −12.2)	−11.1 (−13.6, −9.3)	−14.9 (−17.3, −12.5)	**<0.001**
LV GLS, %	−13.9 (−15.5, −12.1)	−9.3 (−12.2, −8.3)	−14.3 (−15.5, −12.6)	**<0.001**
LGE, number of AHA segments	69 (49%)	8 (47%)	61 (49%)	0.87
LGE septal	32 (23%)	9 (53%)	23 (19%)	**0.004**
LGE mass, %	9 (4, 13)	11 (5, 16)	8 (4, 13)	0.35
LGE mass, g	7 (3, 11)	8 (5, 12)	6 (3, 11)	0.24
Pericardial involvement	34 (24%)	3 (18%)	31 (25%)	0.76
T2 total, ms	59.3 (55.8, 63.1)	61.4 (59.5, 63.9)	59.0 (55.6, 62.6)	**0.047**

^1^ Median (IQR) or frequency (%). ^2^ Fisher’s exact test; Wilcoxon rank sum test; Pearson’s chi-squared test.

**Table 2:** Univariable Cox proportional hazards regression analysis of clinical and CMR characteristics for prediction of ventricular arrhythmic events. Abbreviations as in Table 1. *p*-values highlighted in bold represent statistically significant values.

Variable	Hazard Ratio (95% CI)	*p*-Value
Gender	1.4 (0.39–4.7)	0.63
Age	1.1 (1–1.1)	**<0.001**
Height	1 (0.94–1.1)	0.89
Weight	1 (1–1.1)	**0.017**
BSA	3 (0.32–29)	0.33
Hypertension	3.5 (1.3–9.3)	**0.01**
Dyslipidemia	4.2 (1.5–12)	**0.0072**
Obesity	4 (1.4–11)	**0.0096**
Current or previous smoking	0.8 (0.18–3.5)	0.77
Diabetes mellitus	7.2 (2.1–25)	**0.0021**
Family history of coronary disease	1.1 (0.35–3.3)	0.9
Chest pain	0.1 (0.039–0.26)	**<0.001**
Heart failure	6 (1.9–18)	**0.002**
Arrhythmias	23 (8.8–63)	**<0.001**
Reservoir	0.9 (0.86–0.94)	**<0.001**
Conduit	0.87 (0.82–0.93)	**<0.001**
Booster	0.88 (0.79–0.97)	**0.012**
LVEF	0.93 (0.89–0.96)	**<0.001**
LV EDV/BSA	1 (1–1)	**<0.001**
LV ESV/BSA	1 (1–1)	**0.0025**
LV SV/BSA	0.96 (0.91–1)	0.12
RVEF	0.96 (0.89–1)	0.19
RV EDV/BSA	0.99 (0.97–1)	0.54
RV ESV/BSA	0.98 (0.93–1)	0.33
RV SV	0.96 (0.92–1)	0.093
LV GRS	0.95 (0.92–0.98)	**0.0012**
LV GCS	1.2 (1.1–1.3)	**<0.001**
LV GLS	1.4 (1.2–1.6)	**<0.001**
LGE, number of AHA segments	1.1 (0.41–2.8)	0.88
LGE septal	5.1 (2–13)	**<0.001**
LGE mass, %	1 (0.98–1.1)	0.24
LGE mass, g	1 (0.99–1.1)	0.11
Pericardial involvement	0.64 (0.18–2.2)	0.48
T2 total	1.1 (0.99–1.2)	0.083

**Table 3:** Multivariable Cox proportional hazards regression analysis. Models are incrementally adjusted with demographics ^1^, cardiovascular risk factors ^2^, LVEF ^3^, and LGE septal ^4^. *p*-values in bold are significant. Abbreviations as in Table 1.

	Multivariable Analysis	
	Hazard Ratio (95% CI)	*p*-Value
Adjusted for sex and age ^1^		
Reservoir	0.92 (0.87–0.97)	**0.002**
Booster	0.85 (0.77–0.94)	**0.002**
Conduit	0.91 (0.84–0.99)	**0.03**
LV GCS	1.25 (1.11–1.40)	**<0.001**
LV GRS	0.93 (0.89–0.97)	**<0.001**
LV GLS	1.25 (1.08–1.43)	**0.002**
+ cardiovascular risk factors ^2^		
Reservoir	0.91 (0.87–0.96)	**<0.001**
Booster	0.84 (0.75–0.93)	**0.001**
Conduit	0.89 (0.82–0.97)	**0.007**
LV GCS	1.44 (1.20–1.72)	**<0.001**
LV GRS	0.87 (0.81–0.94)	**<0.001**
LV GLS	1.37 (1.16–1.61)	**<0.001**
+ LVEF ^3^		
Reservoir	0.93 (0.88–0.99)	**0.03**
Booster	0.89 (0.78–1.00)	**0.049**
Conduit	0.93 (0.85–1.01)	0.1
LV GCS	1.39 (1.12–1.73)	**0.003**
LV GRS	0.89 (0.82–0.97)	**0.006**
LV GLS	1.27 (1.03–1.56)	**<0.001**
+ LGE septal ^4^		
Reservoir	0.93 (0.87–0.99)	**0.02**
Booster	0.87 (0.76–0.99)	**0.04**
Conduit	0.92 (0.84–1.02)	0.1
LV GCS	1.37 (1.08–1.73)	**0.008**
LV GRS	0.89 (0.80–0.98)	**0.01**
LV GLS	1.26 (1.02–1.55)	**0.03**

## Data Availability

The data underlying this article cannot be shared publicly due to the privacy of the individuals that participated in this study. The data may be shared on reasonable request to the corresponding author.
